# Spectroscopic Profile of Metabolome Dynamics During Rat Cortical Neuronal Differentiation

**DOI:** 10.3390/ijms26168027

**Published:** 2025-08-20

**Authors:** Idália Almeida, Filipa Martins, Brian J. Goodfellow, Alexandra Nunes, Sandra Rebelo

**Affiliations:** 1 Institute of Biomedicine (iBiMED), Department of Medical Sciences, University of Aveiro, 3810-193 Aveiro, Portugal; idalia24@ua.pt (I.A.); samartins@ua.pt (F.M.); 2 CICECO—Aveiro Institute of Materials, Department of Chemistry, University of Aveiro, Campus Universitário de Santiago, 3810-193 Aveiro, Portugal; brian.goodfellow@ua.pt

**Keywords:** FTIR, cortical neurons, proteomics, metabolomics, neuronal differentiation

## Abstract

Neuronal differentiation is a highly dynamic process marked by coordinated biochemical, structural, and metabolic changes. Rat primary cortical neurons are the preferred cell model to study this process as they can maintain their functional attributes, including functional synapses, and simulate the behavior of neuronal cells in vivo. In this study, we employed Fourier transform infrared (FTIR) spectroscopy to monitor the molecular transformations that occur during the differentiation of rat cortical neurons. Partial least squares regression (PLS-R) analysis from the 1800–1500 cm^−1^ region further allows the identification of the spectroscopic profile of early and late differentiation stages, highlighting the technique’s ability to detect subtle molecular changes. Further peak intensity analysis revealed significant changes in the cells’ metabolome during differentiation; it was possible to observe remodeling of protein secondary structures and an increase in protein phosphorylation levels, which can imply activation of signaling pathways essential for neuronal differentiation and maturation. Concomitantly, lipid-associated spectral regions demonstrated increased levels of total lipids, lipid esters, and longer acyl chains and decreased unsaturation levels, alterations that can be linked to membrane expansion throughout neuronal differentiation. These findings underscore FTIR spectroscopy as a valuable tool for studying neuronal differentiation, offering insights into the conformational and metabolic shifts underlying the formation of mature neuronal phenotypes.

## 1. Introduction

Neurons are polarized cells capable of receiving, integrating, and transmitting information to other neurons and organs. There are two distinct types of processes arising from the soma: a single, long axon and several shorter dendrites [1,2,3]. Early in the differentiation process, neurons lack this polarized morphology. Neuritogenesis begins with the emergence of neurites from the cell soma, after which neuronal polarization occurs, establishing a leading process that develops into the axon and processes that become dendrites [4,5,6]. Neuronal differentiation can then be categorized into five stages that have already been translated to days for samples of hippocampal neurons: initially spherical neurons form small projections that will give way to axons and dendrites (stage 1, 6 h of plating); growth cones start extending and forming multiple neurites (stage 2, 12 h); one neurite breaks symmetry as it elongates into the axon (stage 3, 36 h); the other neurites grow into dendrites (stage 4, 4 days in vitro (DIV)); and the dendrites will further mature, forming dendritic spines and synapses (stage 5, 7 DIV) [2,7]. This stepwise progression of neuronal differentiation is closely orchestrated by cytoskeletal dynamics, where the cytoskeleton and the associated proteins play an important role in neuronal differentiation. The growth cones, constituted by filamentous actin (F-actin), microtubules (MTs), and neurofilaments, sense extracellular signals, and as such, adjust the cytoskeleton formation [4,8,9]. The neuronal cytoskeleton is essential for maintaining cell shape and guiding neurite migration and extension [10]. Throughout neuronal polarization, the future axon growth cone undergoes several changes that involve the transformation of the distal domains of the microtubules into a stable neurite shaft, progressing through repeating cycles of protrusion, engorgement, and consolidation [11]. Concurrently, dendrites remain static with a stable actin cytoskeleton [8]. During the protrusion phase, F-actin polymerization drives membrane extension, followed by the engorgement phase where microtubules transport organelles into the growth cone. In the consolidation phase, bundled microtubules stabilize, forming the neurite shaft [8,10]. This process is closely linked to the spatial organization and dynamics of the cytoskeleton. Tubulin heterodimers (comprising α- and β-tubulin) polymerize to form microtubules and are essential for neurite outgrowth [12]. This polymerization involves longitudinal and lateral interactions between tubulin heterodimers, leading to the formation of microtubules [13,14]. Microtubules have a plus end, where dimers are primarily added (polymerization) or removed (depolymerization), and a minus end, which can also undergo depolymerization but is often stabilized in neurons. As a result, microtubule dynamics in neurons are mainly governed by activity at the plus end [10]. During neuronal polarization, the stability of neurite microtubules increases through mechanisms such as enhanced polymerization, suppression of destabilizing factors, bundling, and the stabilization of microtubules. This dynamic is tightly regulated by microtubule-binding proteins (MBPs) such as members of the microtubule-associated protein (MAP) family, which control microtubule organization and dynamics; post-translational modifications contribute to the regulation of microtubule behavior throughout neuritogenesis [8,10,15]. The cell controls the length, stability, number, and organization of cytoskeletal filaments primarily through their interactions with other structures and accessory proteins. This regulation, especially of the actin cytoskeleton, is crucial for neurite outgrowth, axon guidance, and synapse formation during neuronal differentiation [16].

Neurons are characterized by several different features that support their function. For example, they express specific adhesion molecules, which determine synaptic specificity. Furthermore, they acquire specific morphologies and target different regions of the body. Additionally, they form chemical or electric synapses, and they use a range of different neurotransmitters to deliver a signal and express different neurotransmitter receptors to receive and propagate signals. Neurons can secrete different signaling proteins and neurotrophic molecules like the BDNF and NGF, being responsible for neuronal differentiation. To acquire these features, neurons express transcription factors that regulate their structural, molecular, and physiological characteristics and generate their impressive cell type diversity. These transcription factors control different features at different times during development and are thus expressed upon neuronal specification and/or during neuronal differentiation. In essence, neuronal differentiation is a tightly regulated process involving the coordinated action of transcription factors, signaling molecules and pathways, neurotrophic molecules, and environmental cues, leading to mature neurons [17].

Primary neuronal cultures, derived from embryonic or postnatal brain tissues, provide essential models for studying neuronal development and differentiation as they can maintain their properties and mimic neuronal differentiation in vivo [18]. These neuronal culture systems allow researchers to study the differentiation of embryonic brain cells from progenitors to mature neurons and investigate the molecular and cellular processes fundamental to neurogenesis. To study human neuronal differentiation, rodents (particularly mice and rats) are the preferred models used to perform primary neuronal cultures. This is because several processes are remarkably similar in rodent and human brains, namely, their neurogenesis, cellular differentiation, synaptogenesis, and myelination [19]. Despite the positive aspects of primary neuronal cultures, they also have limitations: a short and limited lifespan, loss of neuronal integrity over time, restrained growth capacity, cultures are less easy to transfect, and the existence of ethical considerations [20]. Given the high relevance of this cell model for studying many relevant human cellular processes, including neuritogenesis, our research group has established a protocol to isolate and differentiate rat cortical and hippocampal neurons [21].

The use of the -omics approaches, such as proteomics or metabolomics, enables the identification of biomolecular alterations associated with diseases but also during physiological processes [22,23]. Metabolomics is increasingly being incorporated into biomedical and medical research and drug discovery, aiding in the identification of disease biomarkers, understanding physiological mechanisms, and developing new therapeutic strategies [24,25,26,27]. Metabolomics allows for the comprehensive study of an entire set of biomolecules, like proteins, lipids, carbohydrates, and nucleic acids (the metabolome) present in a cell, tissue, or organism, facilitating their identification and characterization, including their levels, interactions, and functions. The study of the metabolome is important since the identification of changes in these biomolecules allows us to understand the interactions between gene expression, cellular environment, and metabolic processes [28,29].

Fourier transform infrared (FTIR) spectroscopy has, in recent years, emerged as a promising tool for screening biological samples [30]. It has been successfully applied in various fields, including the identification of cancer [23] and neurodegenerative diseases biomarkers [31], characterization of metabolic profile of aging [32], muscular dystrophies [33], and quantification of analytes in biological fluids [34,35], yielding reliable and reproducible results. A major advantage of FTIR spectroscopy is its ability to quickly generate a spectroscopic profile that reflects the characteristic molecular content of a sample, providing valuable insights into the levels of the different biomolecules. Thus, FTIR is an excellent starting point for metabolomic studies, allowing researchers to assess the sample’s behavior and compare spectroscopic profiles across different sample groups [30,32].

In the present manuscript, we hypothesized that FTIR spectroscopy could be used to determine the fingerprinting of the neuronal differentiation process. For this purpose, we used rat primary cortical neurons and FTIR spectroscopy to assess changes in the metabolomic profile of whole cells at different DIVs. Using this approach, we sought to characterize the metabolomic profile of neuronal differentiation from the undifferentiated cells to mature neurons. We observed that the spectroscopic profile of neurons with more DIVs differs from that with fewer DIVs in the 1800–1500 cm^−1^ region. Additionally, we detected an increase in total protein content and phosphorylation levels, along with a shift in protein conformation. During neuronal differentiation, levels of total lipids, acyl chains, and lipid esters increased, while lipid unsaturation levels decreased. A decrease in RNA levels was also observed throughout this process.

## 2. Results

### 2.1. PLS-R Multivariate Analysis of Spectroscopic Profile

**Figure 1:**
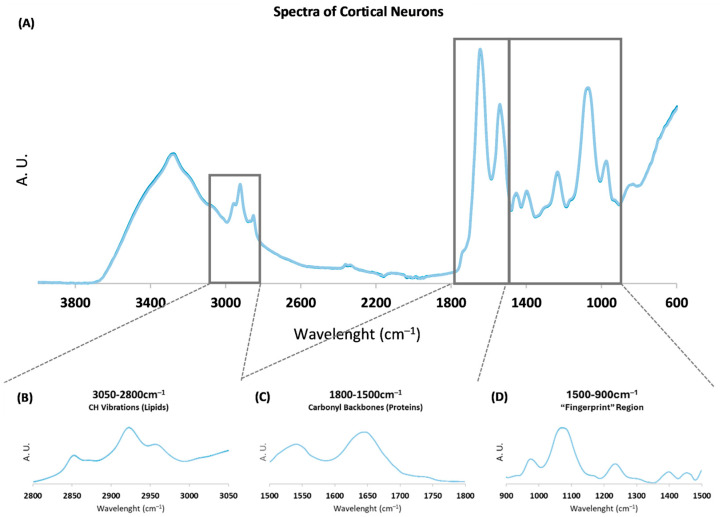
(**A**) Representative Fourier transform infrared (FTIR) spectra of rat cortical neurons in the mid-infrared (4000–600 cm^−1^) region from 2 to 14 days in vitro (DIV). Boxes represent spectral regions used for statistical analysis and peak area analysis. Area-normalized spectra from (**B**) 3050–2800 cm^−1^, with CH vibrations mainly from lipids; (**C**) 1800–1500 cm^−1^, mainly from carbonyl backbones from proteins; and (**D**) 1500–900 cm^−1^, the “fingerprint” region. A. U.: Arbitrary units.

To assess the spectroscopic profile of neuronal differentiation, FTIR spectra from cortical neurons at different DIVs were acquired, namely 2, 4, 6, 8, 10, 12, and 14 DIVs. Figure 1 shows representative baseline-corrected and area-normalized FTIR spectra of cortical neurons.

**Figure 2:**
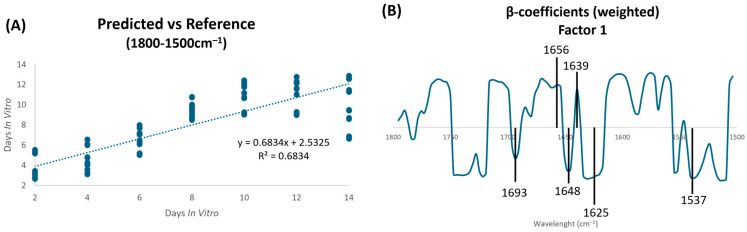
PLS-R multivariate analysis of rat cortical neurons in the 1800–1500 cm^−1^ region. (**A**) PLS-R predicted vs. reference plot of factor 1 of the second derivative spectra of cortical neurons. (**B**) β-coefficients plots of factor 1. The positive peaks characterize cells with more DIVs, and the negative peaks characterize cells with fewer DIVs.

To further investigate the impact of neuronal differentiation on protein conformation, a partial least squares regression (PLS-R) was applied to analyze the spectral region of 1800–1500 cm^−1^. The 1800–1500 cm^−1^ region encompasses the peak at 1740 cm^−1^ that is assigned to ester groups of lipids and the 1700–1500 cm^−1^ sub-region that corresponds to vibrations of carbonyl backbones of proteins and includes the amide I and amide II spectroscopic signals of proteins. The amide I peak is particularly responsive to changes in protein secondary structure, enabling the detection of conformational alterations in cortical neurons during differentiation [32,36]. PLS-R demonstrated a moderate to strong positive linear correlation between the spectral profile and DIV [37], with a correlation value of calibration of 0.68 (Figure 2A). This result was further supported by internal cross-validation, which also yielded a correlation coefficient of 0.68 (Table 1).

The β-coefficients of Factor 1 allow tracing the spectroscopic profile characteristic of 2 DIV and 14 DIV (Figure 2B). The peaks responsible for this discrimination are located at 1656 cm^−1^, which is assigned for β-turns, and at 1639 cm^−1^, which is assigned for parallel β-sheet. These peaks are associated with cortical neurons with more DIVs. The peaks at 1693 cm^−1^, 1648 cm^−1^, 1625 cm^−1^, and 1537 cm^−1^, which are assigned to antiparallel β-sheet, α-helix, intermolecular β-sheet structures, and amide II groups, respectively, characterize cortical neurons with less DIVs.

### 2.2. Peak Intensity Analysis Related to Protein Conformation

**Figure 3:**
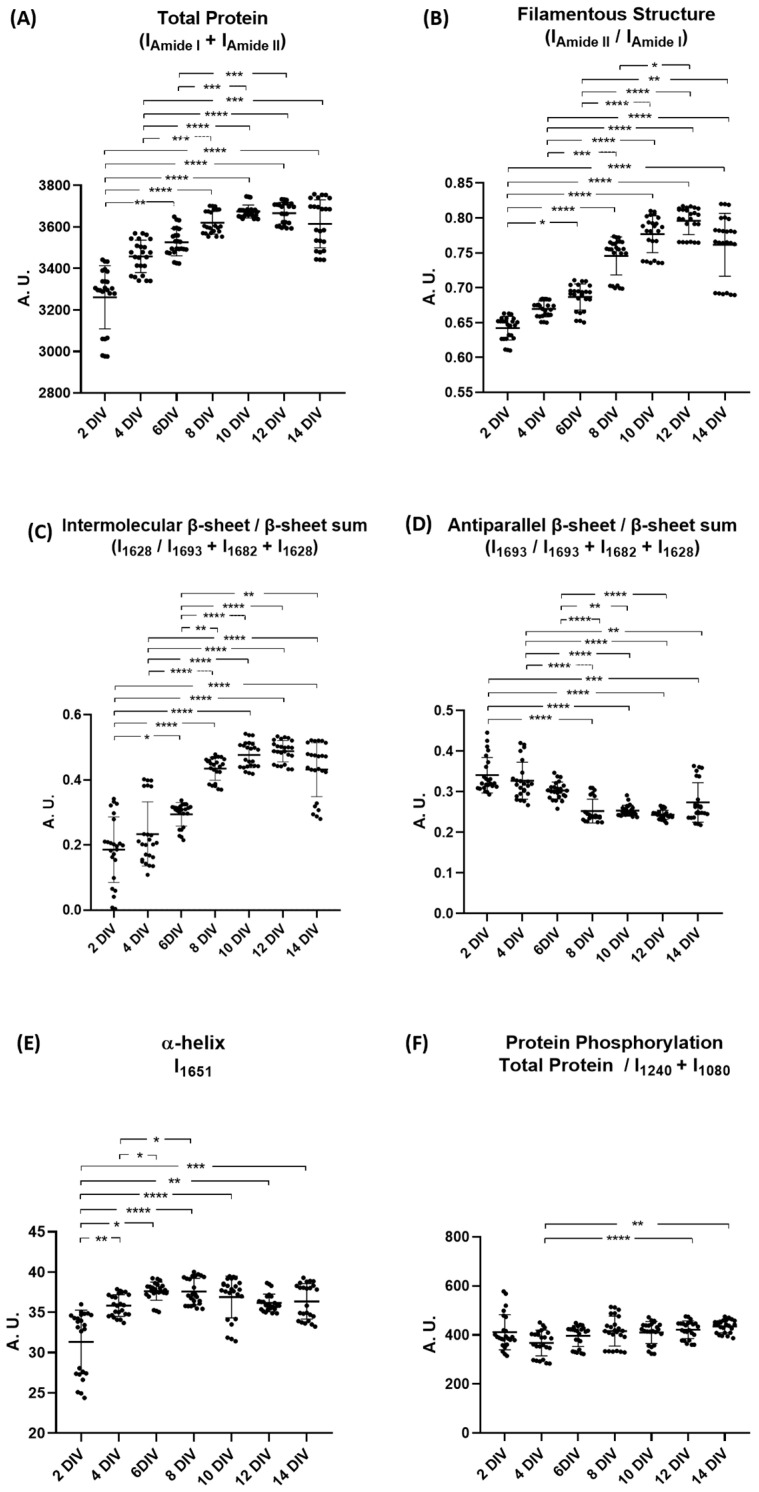
Analysis of peak intensities regarding neuronal differentiation at 2, 4, 6, 8, 10, 12, and 14 DIVs. (**A**) Total protein levels (I_AmideI_ + I_AmideII_, using non-derived spectra); (**B**) Filamentous structures (ratio I_AmideII_/I_AmideI_, using non-derived spectra); (**C**) Ratio of intermolecular β-sheets/β-sheets’ sum (I_1628_/I_1693_ + I_1682_ + I_1628_); (**D**) Ratio of antiparallel β-sheet/β-sheets’ sum (I_1693_/I_1693_ + I_1682_ + I_1628_); (**E**) Peak intensity of α-helix (I_1651_); (**F**) Ratio of protein phosphorylation (total protein levels/I_1240_ + I_1080_). All ratios were calculated using respective peak intensities. Data are expressed as mean ± SD. * *p* < 0.05; ** *p* < 0.01; *** *p* < 0.001; **** *p* < 0.0001. A. U.: Arbitrary units. DIV: Days in vitro. I: Intensity.

Peak intensity analysis was conducted (i) to confirm the protein structural changes upon differentiation and (ii) to identify changes in the metabolomic profile of cortical neurons differentiation. First, we focused on the analysis of ratios and peak intensities that provided information about variations in protein conformation of cortical neurons. We utilized the second derivative spectra and spectra that were normalized. The results of this analysis are shown in Figure 3. Cortical neurons at different DIVs, namely 2, 4, 6, 8, 10, 12, and 14 DIVs, were compared between each other. By summing the amide I and amide II peaks, we observed, that when compared to 2 DIV, total protein levels increased significantly from 6 DIV to 14 DIV, although these levels stabilized after 10 DIV. A similar trend was observed when comparing to 4 DIV and 6 DIV; however, in the case of 6 DIV, the increase was significant up to 10 DIV, but not at 14 DIV (Figure 3A). A similar trend can be observed for the filamentous structures (Figure 3B) and intermolecular β-sheets (Figure 3C); the exception being 6 DIV, which significantly increases when comparing from 8 to 14 DIV for both, and in the case of the filamentous structures, where there is also a significant increase from 8 to 12 DIV. The levels of antiparallel β-sheets significantly decreased over time. A statistically significant reduction was observed when comparing 2 DIV from 8 DIV to 14 DIV and similarly when comparing 4 DIV from 8 DIV to 14 DIV. In addition, 6 DIV showed a significant decrease when comparing from 8 DIV to 12 DIV (Figure 3D). The peak intensity corresponding to α-helix structures (Figure 3E) increased significantly throughout the differentiation process. Regarding protein phosphorylation, the levels remain constant throughout neuronal differentiation, except at 12 and 14 DIVs, where a significant increase in phosphorylation is observed compared to 4 DIV (Figure 3F).

### 2.3. Peak Intensity Analysis of Spectroscopic Signals Related to Lipids and Nucleic Acids

**Figure 4:**
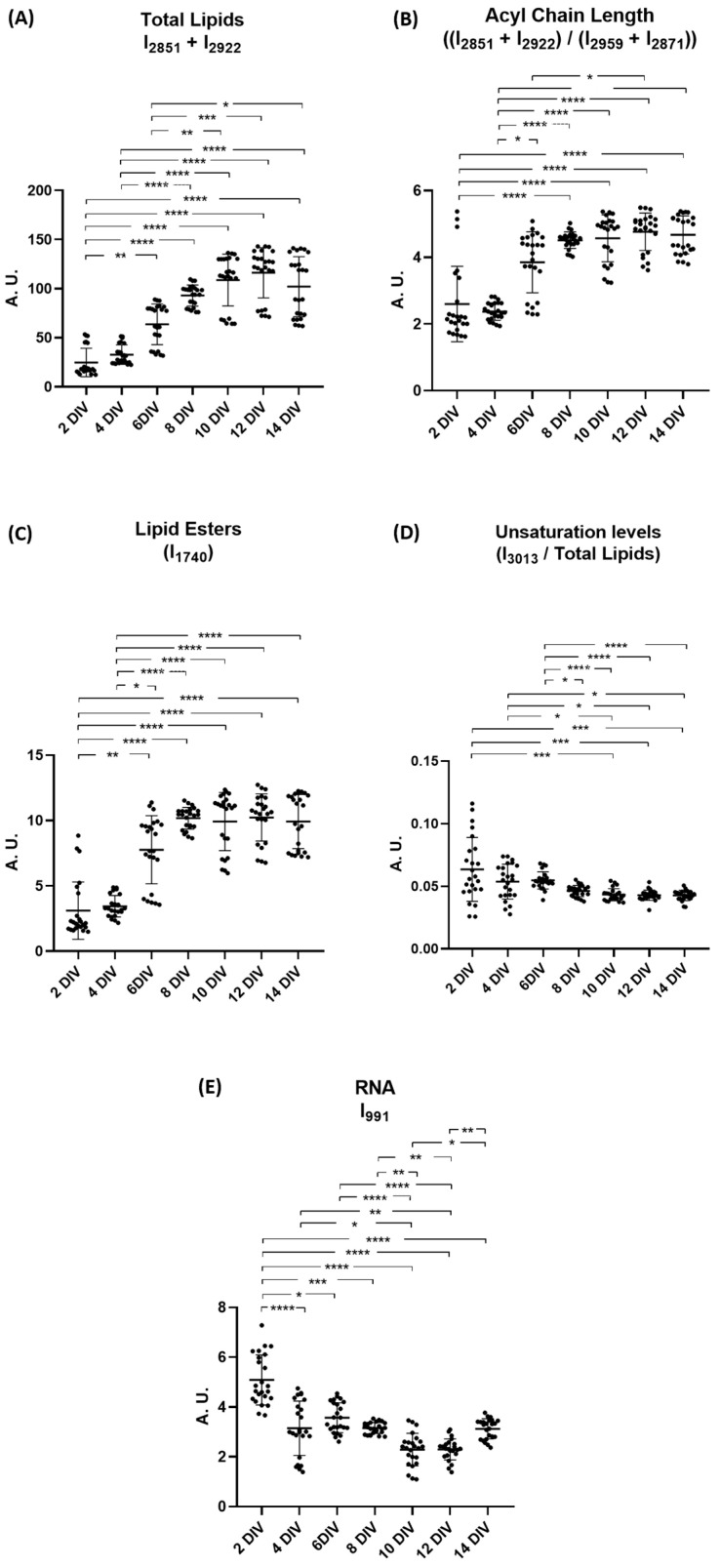
Analysis of peak intensities of rat cortical neurons at 2, 4, 6, 8, 10, 12, and 14 DIVs. (**A**) Total lipid levels (CH_2_ groups) (I_2851_ + I_2922_); (**B**) Acyl chain length ratio ((I_2851_ + I_2922_)/(I_2959_ + I_2871_); (**C**) Peak intensity of lipid esters (I_1740_); (**D**) Unsaturation levels (I_3013_/Total Lipids); (**E**) Peak intensity of C–O stretching from RNA ribose chain (I_991_). All ratios were calculated using respective peak intensities. Data are expressed as mean ± SD. * *p* < 0.05; ** *p* < 0.01; *** *p* < 0.001; **** *p* < 0.0001. A. U.: Arbitrary units. DIV: Days in vitro. I: Intensity.

The next step was the analysis of the intensity of peaks associated with functional groups of lipids and nucleic acids. This time, only the intensity of the peaks of second derivative spectra were used to calculate the ratios; again, all neurons DIVs were compared with 2 DIV. Results are shown in Figure 4. Regarding the lipid functional groups, there is a significant increase in the amount of total lipids (CH_2_ groups) comparing 2 DIV from 8 DIV to 14 DIV. Also, the increase is significant comparing 4 DIV from 8 DIV to 14 DIV and comparing 6 DIV from 10 DIV to 14 DIV (Figure 4A). Moreover, acyl chain lengths significantly increase comparing 2 DIV to time points from 8 DIV to 14 DIV. A similar significant increase was observed when comparing 4 DIV from 6 DIV to 14 DIV as well as 6 DIV to 12 DIV (Figure 4B). The peak intensity corresponding to lipid esters significantly increases from 2 DIV and 4 DIV until 6 DIV to 14 DIV (Figure 4C). The unsaturation levels suffered a significant decreased from both 2 DIV and 4 DIV to 10 DIV, 12 DIV, and 14 DIV and also from 6 DIV and 8 DIV to 14 DIV (Figure 4D). For RNA peak intensity, we observed a significant decrease throughout all days of neuronal differentiation, despite the variation not being linear (Figure 4E).

## 3. Discussion

The analysis of the spectroscopic profile of rat cortical neurons during differentiation and the identification of proteomic and metabolomic alterations that occur during this process is an innovative approach and of paramount importance. In this study, ATR-FTIR spectroscopy was employed to characterize the biomolecular profiles of rat cortical neurons differentiation. Primary cortical neurons derived from embryonic tissues are preferred models for the study of neuronal differentiation since all environmental conditions are created to mimic neuronal differentiation and maturation in vivo [18,19]. In vitro neuronal differentiation produces mature neurons capable of forming functional synapses [38]. This cellular model can be used to study disease mechanisms, for example, by expressing a central gene implicated in Alzheimer’s disease, such as the wild-type or mutant form of amyloid precursor protein (APP) [39,40]. Of note, these cultures are mainly composed of cortical neurons. To guarantee that these cultures are enriched in cortical neurons, 5-fluoro-2′-deoxyuridine (FUdR) supplementation was added to the culture medium to inhibit the proliferation of glial cells, reducing their contribution to the analysis [41]. Previous studies have reported proteomic analyses of rat hippocampal neurons [42] and SH-SY5Y cell differentiation [43,44]. Additionally, FTIR microspectroscopy has been used to investigate the differentiation of mouse embryonic stem cells into embryonic stem-derived neural cells [45]. However, to the best of our knowledge, none of these studies used FTIR spectroscopy to characterize metabolomic profiles specifically during cortical neuron differentiation, making this manuscript pioneering. Although key differentiation pathways are conserved, species-specific metabolic differences should be considered. Future studies using human-derived neuronal models are needed to confirm the translatability of our findings.

Multivariate analysis offers an effective and reliable means of interpreting complex data like the spectral profile of neuronal differentiation [46,47]. In fact, PLS-R allowed for discriminating neuronal spectra into distinct groups corresponding to different differentiation stages and to identify the most significant spectral differences among groups [48]. FTIR analysis of the 1800–1500 cm^−1^ region reveals variations in protein conformations associated with neuronal differentiation, showing that the protein profiles of cells with higher DIVs have are different from those with fewer DIVs. Neurons with more DIVs are characterized by β-turns and parallel β-sheet structures, and neurons with fewer DIVs by antiparallel and intermolecular β-sheet and α-helix structures. These findings confirm that, during the process of neuronal differentiation, proteins acquire different structures until they form a stable network. Based on these results, we can say that it is possible to follow the differentiation of cells with spectroscopy and proper multivariate analysis tools, and a moderate to strong correlation is found between the spectroscopic profiles of the cells during the differentiation process [37]. A more detailed analysis was conducted to investigate both protein conformational changes and alterations in the metabolomic profile.

Intensities of peaks assigned to proteins, lipids, and RNA functional groups were calculated. During neuronal differentiation, the total protein content of cells increases significantly, possibly marking the transition from undifferentiated cells to mature neurons. This reflects extensive neuronal growth, including neurite extension, which requires the synthesis of large amounts of proteins [49]. Proteomic studies reveal that nearly one-third of quantified proteins change expression by more than two-fold during neuronal differentiation [42]. Also, similar studies using cortical neurons and SH-SY5Y cells reveal an increase in proteins involved in neuronal differentiation, like neuronal projections, and synaptic function as differentiation proceeds [44,50].

An increase in filamentous structures was also observed throughout the differentiation process. The increase in filamentous structures likely corresponds to the formation of microtubules, neurofilaments, and actin microfilaments, which drive cytoskeletal reorganization and neuronal polarization [51]. During differentiation, tubulin dimers assemble into microtubules, forming networks that stabilize the neurite structure [51]. At the same time, actin filaments, especially in growth cones, drive cytoskeletal rearrangements, supporting neurite extension and guidance [8,9].

The increase in intermolecular β-sheets during neuronal differentiation could reflect the assembly of diverse protein families, signaling a shift toward a more organized and specialized neuronal architecture. These conformations play a crucial role in biomolecular recognition and contribute to protein quaternary structure and protein–protein interactions [52,53]. During neuronal differentiation, there is a decrease in antiparallel β-sheet structures, which are known to be less stable and often associated with transition intermediates [54]. This structural transition may imply the formation of more stable and functional neuronal architectures and allows for greater structural flexibility, enhancing dynamic protein interactions, which are essential for neuronal maturation and specialization. In support of this observation, we saw that α-helix structures increase up to 8 DIV. During neuronal differentiation, extensive remodeling of protein conformations occurs, driven by environmental changes and functional demands. The membrane environment favors α-helix stabilization in the transmembrane domains of neuronal proteins [55].

Results show an increase in protein phosphorylation at 14 DIV, highlighting its pivotal role in neuronal differentiation. Protein phosphorylation is a highly dynamic and tightly regulated process that is a central regulatory mechanism during neuronal development [56]. Phosphorylation is particularly critical in the early phases of differentiation, regulating axon and dendrite formation as well as synaptic signaling pathways [44,57]. It also controls cytoskeletal remodeling, crucial for neurite extension and axonal guidance.

Tau and MAP2 are microtubule-associated proteins whose phosphorylation dynamically regulates neuronal differentiation. Early in development, tau is highly phosphorylated to promote microtubule remodeling and cytoskeleton plasticity; as neurons mature, tau phosphorylation decreases [58]. MAP2, primarily found in dendrites, undergoes phosphorylation changes that control neurite outgrowth [59]. Phosphorylation of both proteins enables cytoskeletal flexibility during differentiation. The MAPK/ERK pathway, activated by growth factors, sequentially triggers ERK1/2. ERK can then activate cytosolic pathways or enter the nucleus to activate different genes. This signaling cascade plays a key role in the maturation process and synaptogenesis and guides neural progenitor cells toward neuronal fates [60]. PI3K activation leads to Akt phosphorylation, and the PI3K/Akt pathway promotes metabolism, cell survival, cell cycle, and protein synthesis, ultimately enhancing neuronal plasticity [61].

Changes in the lipid spectroscopic profile during neuronal differentiation were also investigated, and a clear increase in total lipid levels was observed. This increase reflects the high demand for membrane synthesis as neurons extend axons and dendrites and form synapses. This demand is higher for phospholipids, the main components of plasma membranes [62,63]. As neurogenesis occurs, there is enhanced lipogenesis, ensuring a sufficient lipid supply for several cellular needs [64]. Additionally, lipids such as sphingolipids play signaling roles, modulating neuronal growth, differentiation, and synaptic plasticity associated with the differentiation process [65].

An increase in acyl chain length was also observed during neuronal differentiation. This increase could imply the acyl chain elongation of lipids [66], which is crucial for modulating membrane characteristics such as thickness, fluidity, protein–lipid interactions, and hydrophobic matching with transmembrane proteins [67], all of which impact neuronal structure and signaling. Moreover, long-chain fatty acids are key for the formation of lipid rafts, which help organize neuronal polarity and are involved in intracellular signaling. Similarly, long-chain ceramides enhance lipid bilayer order, reducing fluidity and stabilizing the membrane [66].

Lipid esters levels also increase throughout differentiation. This increase supports rapid neuronal growth, neurite extension, and synapse formation. Developing neurons accumulate lipid droplets rich in triglycerides and cholesterol esters, serving as fatty acid reservoirs for membrane synthesis and remodeling [68,69]. Lipid esters stored in droplets can be used to generate the phospholipids and other lipids necessary for membrane expansion in neurite outgrowth [63,69]. As differentiation progresses, there is an increase in the storage of lipids in neurons, reflected by higher levels of triacylglycerols [70]. These lipid esters are essential to provide structural components, ensuring a sufficient supply for membrane biogenesis and neuronal maturation.

A decrease in lipid unsaturation levels during neuronal differentiation was also observed. Polyunsaturated fatty acids (PUFAs), such as docosahexaenoic acid (DHA), play a crucial role in early neuronal development by enhancing membrane fluidity and flexibility, which supports processes like neurite outgrowth and synaptogenesis [71,72,73]. However, as neurons mature, a shift in lipid composition seems to occur. This shift may involve a reduction in highly unsaturated lipids, particularly n-6 PUFAs, which in excessive amounts can disrupt electrophysiological activity, leading to asynchronous neuronal firing and excitatory–inhibitory imbalances [74]. Additionally, PUFAs are highly susceptible to lipid peroxidation, increasing the risk of oxidative damage, which threatens neuronal survival and function [75]. Therefore, the reduction in unsaturation likely represents a protective adaptation during maturation, promoting membrane stability and reducing oxidative vulnerability. This compositional adjustment supports the long-term maintenance of functional neuronal circuits.

Lipids play a central and multifaceted role in neuronal differentiation. Phospholipids, the primary components of cell membranes, are essential for maintaining membrane structure [63]. Phosphatidylinositol serves as a precursor for the signaling molecule phosphatidylinositol 4,5-bisphosphate, which is involved in neurite outgrowth and cytoskeletal organization [76,77]. Sphingolipids are key constituents of lipid rafts, specialized membrane microdomains that are critical for neuronal differentiation [78]. Cholesterol contributes to membrane fluidity and the structural organization of lipid rafts, thereby influencing axon formation, neurite extension, and synapse formation [78].

The observed protein and lipid changes are functionally significant in promoting neuronal maturation and enabling synaptic plasticity. The dynamic regulation of key cytoskeletal proteins supports neurite formation and neuronal polarization [51]. The upregulation of synaptic proteins reflects the progressive formation, stabilization, and functional refinement of synaptic contacts [79]. Lipid remodeling, particularly within lipid rafts, facilitates critical aspects of membrane dynamics. These include synapse organization [80], dendritic spine maturation, and membrane trafficking [78]. Together, these protein and lipid alterations orchestrate the transition from immature neurons with silent or unstable connections to fully integrated neuronal networks capable of efficient signal transmission and adaptive plasticity.

The RNA levels decreased during neuronal differentiation, which is likely due to large-scale transcriptional repression of genes involved in cell cycle regulation, RNA processing, and chromatin remodeling, which are actively suppressed [81,82]. The neuronal development includes a global downregulation of transcripts, with over half of significantly regulated genes being downregulated, marking it as the most prominent transcriptional shift during differentiation [82]. Notably, the variation in gene expression is not primarily due to translation, but rather to a transcriptional activity [83]. Various types of nucleic acids play essential roles in neuronal differentiation. Messenger RNAs (mRNAs) encode transcription factors that are key to activating neuronal gene expression programs, thereby guiding neural progenitors toward maturation [84]. MicroRNAs (miRNAs) also contribute to neuronal differentiation. For example, miR-134 is a brain-specific microRNA whose expression increases during embryonic differentiation, where it promotes neural progenitor proliferation [85]. In dendrites, miR-134 controls dendritic spine morphology and synaptic plasticity [86]. Non-coding RNAs (ncRNAs) contribute to the epigenetic regulation of neuronal genes by interacting with chromatin-modifying complexes. Although the structure of DNA remains unchanged, neuronal differentiation is accompanied by epigenetic modifications at specific gene loci, which modulate access to the transcriptional machinery [84].

Inflammation, oxidative stress, and metabolic dysfunction influence neuronal differentiation under pathological conditions. Compounds like lobetyolin, forsythoside B, gypenoside-14, and GIP promote neuronal survival and differentiation by modulating key signaling pathways (e.g., AKT, ERK, Nrf2, PI3K/AKT) and reducing apoptosis and oxidative damage [87,88,89,90]. Under hypoxia, reduced CIRP interaction with GluR1 impairs synaptic plasticity, suggesting its dysfunction may hinder neuronal maturation. These findings highlight the importance of targeting stress-related pathways to support neurodevelopment and recovery [91].

The use of FTIR spectroscopy allows us to visualize an overall picture of metabolomic alterations during neuronal differentiation. This technique enables the simultaneous observation of differences in lipid profiles, protein conformations, post-translational modifications, and nucleic acid alterations, all with a single method. The major limitation of using FTIR spectroscopy is that it does not allow for the identification of specific molecules, but it rather detects functional groups common to classes of biomolecules. This restricts our ability to draw definitive molecular conclusions, as the observed spectral changes can only be interpreted as indicative of shifts in broader biochemical classes. To overcome this, future studies incorporating proteomic and lipidomic approaches, like mass spectrometry or nuclear magnetic resonance, are necessary to identify specific proteins and lipids that undergo structural changes during neuronal differentiation. Nevertheless, this study establishes a physiological baseline that is essential for the comparison with pathological conditions and opens the door for future investigations into disease-related biochemical alterations.

## 4. Materials and Methods

### 4.1. Neuronal Primary Culture Establishment

The Institute of Biomedicine (iBiMED) from the University of Aveiro has an animal facility that is licensed by the competent Portuguese national authority (DGAV). It is run by a dedicated veterinary and technician. All experimental procedures were performed in accordance with the European Communities Council Directive (2010/63/EU) on animal experiments under a protocol approved and supervised by the Institutional (Medical Sciences Department, University of Aveiro) Animal Welfare Body (approval number 01/2018). Rat primary cortical cultures were established at embryonic day 18 (E18). Essentially, at E18 pregnant rats were euthanized by decapitation, followed by rat embryo decapitation and cortex tissue isolation. The tissue was subsequently dissociated with 0.45 mg/mL trypsin. Then, the cells were plated onto poly-D-lysine-coated 100 mm petri dishes at a density of 8 × 10^6^ cells in B27-supplemented neurobasal medium (Gibco, Thermo Fisher Scientific, Waltham, MA, USA), a serum-free medium. The medium was further supplemented with 0.5 mM of glutamine, 10,000 units/mL of penicillin, and 10,000 μg/mL of streptomycin (complete medium) as described in [92]. Cultures were maintained in a controlled environment of 5% CO_2_ at 37 °C. Upon 3 days in culture (3 DIV), FUdR was incorporated into the medium to avoid the proliferation of glial cells [41]. Of note, FUdR has been used as a mitotic inhibitor in astrocytes and glia proliferation [41]. Therefore, upon adding FUdR, the glial cells do not proliferate. Further, the presence of glial cells in these neuronal cultures is very low compared to neurons, meaning that the cultures are mainly composed of neurons. The rat cortical neurons were collected every 2 days up to 14 DIV (2 DIV-14 DIV) for subsequent analysis using FTIR.

### 4.2. Cell Collection for FTIR Analysis

For the collection of rat cortical neurons, they were dissociated from cell culture dishes using an optimized trypsinization procedure. Briefly, neurons were washed with PBS twice and trypsinized with 0.05% trypsin-EDTA (Gibco, Thermo Fisher Scientific, Waltham, MA, USA) for 6–8 min. The cells were resuspended in 10 mL of complete medium, as described above. Then, the cells were centrifuged at 1000 rpm at room temperature (RT) and resuspended again in 6 mL of complete medium. Trypan blue was mixed in an aliquot of the suspended cells, and the number of viable and non-viable cells was counted using a hemocytometer, as previously described [33,93]. The cell suspension was subsequently centrifuged for 2 min at 1000 rpm at RT. Cells were washed with PBS, and aliquots with 250,000 cells were centrifuged again (2 min, 1000 rpm, RT). Finally, PBS was removed, and the cells were frozen at −80 °C until FTIR analysis. A total of 250,000 cells was the optimized number required to produce high-quality spectra.

### 4.3. FTIR Spectra Measurements

FTIR spectra of rat cortical neurons from 2 DIV to 14 DIV were acquired using a Bruker Alpha Platinum spectrometer (Bruker©, Billerica, MA, USA) equipped with an attenuated total reflectance (ATR) crystal in the mid-infrared range (4000–600 cm^−1^). Spectral acquisition was conducted with OPUS software version 7.0 (Bruker©, Billerica, MA, USA). The measurements were performed at a spectral resolution of 8 cm^−1^, with 64 co-added scans, and under controlled environmental conditions (temperature: 23 °C, relative humidity: 35%). Cell pellets were placed centrally on the ATR crystal and allowed to air dry. The drying process was monitored in real-time using OPUS software, and spectral acquisition began once no further changes in the spectral profile were observed. A background spectrum was recorded against air, and the ATR crystal was cleaned with 70% ethanol and dH_2_O between measurements.

### 4.4. Spectra Pre-Processing

FTIR spectra were exported in the OPUS format and imported into The Unscrambler X software (version 10.4, AspenTech, Bedford, MA, USA) for further analysis. Each experimental condition was analyzed using eight independent biological replicates, with three technical replicates per condition. Each spectrum was visually inspected to identify and exclude those with abnormal profiles, primarily due to inadequate sample drying. Spectra exhibiting such anomalies were remeasured to ensure data quality. Subsequently, principal component analysis (PCA) was conducted to detect and remove outliers, as previously described [30]. Spectral data were pre-processed using baseline correction, and the respective regions were area normalized. The normalized spectra were further processed by calculating the second derivative using the Savitzky–Golay algorithm with three smoothing points. The second derivative approach was employed to deconvolute overlapping bands, enhancing spectral resolution and minimizing variability among replicates. Pre-processed spectra were then subjected to PLS-R, as well as targeted analysis of specific peak intensities.

### 4.5. PLS-R Multivariate Analysis

Spectra were divided. Three main spectral regions were analyzed: 3050–2800 cm^−1^, where CH vibrations mainly from lipids are found, 1800–1500 cm^−1^, where carbonyl backbones from proteins are noted, and 1500–900 cm^−1^, which corresponds to the “fingerprint” region. In this study, PLS-R supervised multivariate analysis was performed on the 1800–1500 cm^−1^ spectral region using second derivative spectra. Random internal cross-validation was performed using the Kernel algorithm, as previously described [32]. The Y matrix corresponded to the DIV, allowing a correlation plot between the spectral profile and DIV of the samples to be obtained. All multivariate analyses were conducted using The Unscrambler X software (version 10.4, AspenTech).

### 4.6. Peak Intensity Analysis

The intensities of spectral bands of cortical neurons from 2 DIV until 14 DIV were calculated using two different approaches. The peak intensities analysis was performed using the peak intensities from second derivative spectra. We studied the peaks assigned to CH of double bonds (3010 cm^−1^), CH_2_ (2851 cm^−1^ and 2922 cm^−1^), and CH_3_ (2959 cm^−1^ and 2871 cm^−1^) groups from lipids, C=O (1733 cm^−1^) groups, protein antiparallel β-sheets (1693 cm^−1^), β-sheets (1682 cm^−1^), intermolecular β-sheets (1628 cm^−1^), PO_2_^−^ groups (1240 cm^−1^ and 1080 cm^−1^), and C–O stretching from the RNA ribose chain (991 cm^−1^) [32,36]. To calculate the ratio of filamentous structures, the total protein content, and the protein phosphorylation, non-derivative spectra were used to extract the intensity values of the amide I and amide II peaks.

### 4.7. Statistical Analysis

Statistical analysis of FTIR peak intensities was conducted using GraphPad Prism 8.0 (GraphPad Software, La Jolla, CA, USA). Comparisons of FTIR peak intensity levels across cortical neurons at 2, 4, 6, 8, 10, 12, and 14 DIV were performed using non-parametric Kruskal–Wallis’ test followed by Dunn’s multiple comparisons test due to the non-normal distribution of the data. Data are presented as mean ± standard deviation, with statistical significance at *p* < 0.05.

## 5. Conclusions

FTIR spectroscopy proved to be a valuable tool for characterizing the biochemical transitions during neuronal differentiation. Analysis of the amide I and II regions revealed distinct spectral signatures associated with different stages of differentiation, allowing for the discrimination of samples based on their maturation state. Our findings provide comprehensive evidence that neuronal differentiation involves profound molecular and structural remodeling of both protein and lipid components. At the protein level, we observed an overall increase in total protein content, accompanied by conformational transitions, that reflects enhanced protein–protein interactions and the establishment of functional neuronal architecture. Concurrently, increased phosphorylation levels support key regulatory pathways essential for neuronal maturation. Lipidomic shifts further highlight the metabolic adaptation of differentiating neurons. The increase in total lipids, lipid esters, and acyl chain length and the decrease in unsaturation levels are consistent with the biosynthetic demands of membrane expansion, neurite outgrowth, and the formation of signaling microdomains. The decrease in RNA levels is consistent with the transcriptional repression of genes unrelated to neuronal differentiation. Altogether, this study underscores the utility of FTIR spectroscopy as a sensitive approach for probing the complex molecular processes underlying neuronal development and offers insight into the structural evolution that accompanies the acquisition of a mature neuronal phenotype.

## Figures and Tables

**Table 1:** Parameters of the PLS-R classification model of factor 1 for the 1800–1500 cm^−1^ spectroscopic region for rat cortical neurons.

	Calibration	Validation
**Correlation**	0.683	0.677
**RMSEC/RMSECV**	2.251	2.283

## Data Availability

The original contributions presented in this study are included in the article. Further inquiries can be directed to the corresponding authors.
